# A Treatise on the Rational Treatment of Stricture of the Urethra

**Published:** 1846-01

**Authors:** 


I. Traité des Angusties ou Rétrécissements, leur Traitment Rationel. Par le Dr. *Leroy D'Etiolles.* 8vo. pp. 488. Paris, 1845.

A Treatise on the Rational Treatment of Stricture of the Urethra.

II. Clinique Medico-Chirurgical du Professeur Lallemand. Publieé par *Hermann Kaula.* 1iere Partie. 8vo. pp. 340. Paris, 1845.

The Medico-Chirurgical Clinical Practice of Professor Lallemand. 1st Part, containing Venereal Affections, Stricture, and Diseases of the Prostate.

Although the numerous books relating to Stricture which have been published of late years might seem to have exhausted the subject, the above-named volumes, from the pens of two distinguished French surgeons, contain numerous useful, and some original, observations respecting it. Dr. Leroy's work is indeed a complete treatise upon the nature, consequences and treatment of the disease, and the varieties of means to be adopted are set forth in a masterly and discriminatory manner. M. Lallemand's "Clinique" consists chiefly of cases illustrative of certain points of practice, accompanied by observations, collected from his oral discourses and former published works, by M. Kaula, one of his private pupils. Much of it therefore is a republication, in a more connected form, of what has hitherto appeared at different epochs: and, although we have not always the *ipsissima verba* of the Professor, the publication has his full sanction, and its appearance, as the first part of a complete collection of M. Lallemand's observations, is the more interesting just now, inasmuch as his professional career may be considered as closed, by his retirement from his professorship and extensive practice at Montpellier. Ill-health and inability to cope with the fatigues of so active a mode of life are assigned as causes for his taking this step: but the witty Jean Raimond cautions his Parisian brethren, among whom M. Lallemand has located himself, that, when he has become possessed of the various professional honours which so justly await him, they may find in him another Sextus Quintus.

It is, however, to M. Leroy's work our chief attention will be directed. He commences it with a few

*Anatomical Considerations.*

It is very important to know the ordinary *Length of the Urethra,* but, concerning this, different surgeons have delivered very different statements. Thus, Boyer says it varies from 11 to 12 inches, while Malgaigne considers 6 inches as the maximum. This latter observer has, however, only measured the canal after death, when the tissues become so contracted, that a female catheter will usually reach the bladder. MM. Velpeau and Civiale, from admeasurements of the living urethra, state $5\frac{1}{2}$ to $6\frac{1}{2}$ inches as its length, although the plates in the work of the latter make it appear much longer. Dr. Leroy agrees with Whateley that the urethra is generally 8 inches, or 22 centimetres* in length, he having found it so in several hundred cases. He has rarely found it so short as 7 inches, but repeatedly as long as 10 or 11 inches. Its length may be much extended by an enlarged prostate in the aged, and by deposition of fatty matter in the tissues surrounding the rectum and neck of the bladder at an earlier period of life. M. Lallemand states that the length of the urethra may vary from 203 to 243 millimetres, and in one patient he observed it even 257 m. The *curvature* of the urethra is also sometimes enormously increased, so that we must not always implicitly rely upon the statements of the anatomist. Although the elevation of the urethra as it enters the neck of the bladder does not in some cases exceed 8 *mill.*, yet, where the prostate is enlarged, or the patient obese, catheters may require to have a curve of even 9 or 10 *centim.* As there is a difference in the entire length of the urethra, so is there in that of its various parts, but in a general way these may be thus measured: the spongy portion $5\frac{1}{2}$ to 6 inches, the membranous 1 inch, and the prostatic portion 12 to 15 lines. M. Lallemand states that the extremes of the length of the urethra from the meatus to the subpubic curve are 150 and 185 millimetres. The length of the penis and development of the prostatic portion will produce great differences, and thus, in a preparation in the Dupuytren Museum, the prostatic portion is observed to be 3 inches long, and the spongy portion only 4 inches. The *diameter* of the urethra in various parts of its course has been also variously stated. The differences, for the most part, disappear when the canal is cut open, so that it is necessary to examine it by injecting wax or plaister, and observing the cast so formed, although, owing to the easy distensibility of the tissues, this forms rather a measure of the elasticity of the canal than of its actual size. Examining it, however, in this way, MM. Leroy and Piégu give results very similar to those published by Sir E. Home. The narrowest and least dilatable portions are the external meatus, the commencement of the membranous portion, and a point corresponding to the anterior third of the spongy portion. However, it is to be observed that, sometimes, the contraction of the meatus occurs only at the orifice, the canal then suddenly dilating; sometimes, there is a second narrowing from 7 to 11 lines lower down, the *fossa navicularis* occupying the space between these; again, the contraction may be continuous for 4 or 5 lines, when there is no fossa; and lastly, the orifice may be funnel-shaped, the narrowest point being found at 3 or 4 lines depth. The vesical orifice of the urethra is in like manner narrowed by the projection of the prostate; but in old cases of stricture this aperture often becomes dilated. The *natural dilatability* of the neck of the bladder is very great in the child, and the ease with which calculi or their fragments become engaged within it, produces the chief obstacle to the performance of litho-

---

* The *Centimetre* is equivalent to rather more than 4 lines—a third of an inch; and the *Millimetre* to $\frac{4}{5}$ of a line. M. Leroy does not always attribute the same value to the millimetre, for while in some places he states 27 millimetres form an inch, in most others he considers a millimetre as equivalent to half a line, when 24 only would be required to form an inch.

trity in the young. When the prostate is hypertrophied this dilatability is less, but even then it will admit a body 6 lines in diameter. In two points the urethra is *dilated* compared to the rest of its course at the bulb, and the centre of the prostate. The bulb is enlarged, especially at the lower wall of the canal; and, as immediately afterwards the urethra becomes narrower and curved, here is the usual site of false passages. The narrow membranous portion resists sudden, but not gradual, dilatation. We will extract some useful remarks on catheterism in reference to this point.

"Surgical works treating of the anatomy of the urethra, rarely fail to state that all the obstacles to catheterism exist at the inferior wall of the urethra, where are united the dilatation of the bulb preceding the sudden contraction of the membranous portion, the projection of the verumontanum, the prostatic dilatation, and the projection of the neck; and the precept, now become trite, is delivered, that the point of the instrument should always follow the upper wall of the canal. * * * * * * The utility of observing this direction at the point of junction between the bulbous and membranous portions of the urethra cannot be doubted; for there is a difference of height of from 4 to 6 millimetres between the one and the other. If the point of the catheter were rested against the bottom of the depression, and force employed to overcome the resistance thus offered, a perforation would be inevitable, and the instrument would pass beneath the membranous portion. Its point must, therefore, be sufficiently raised to avoid the cavity of the bulb; but if, with this object, the upper wall of the urethra is pressed against too firmly, it often happens that the catheter strikes against the sub-pubic ligament, and turns round in the hand of the operator when he attempts to depress the handle. It must not be forgotten that before reaching this ligament, which surrounds it like a collar, the urethra is abandoned by the corpora cavernosa which have separated, and that it is only strengthened anteriorly by the fibrous band which is inserted into the pubis; so that, anterior to the ligament, it possesses a certain laxity which allows it to engage the instrument, and arrest the course of the point, which, after having traversed the portion of the canal surrounded by the corpora cavernosa, passes into the depression formed between them and the sub-pubic ligament. Here false passages are less to be feared than posteriorly, because resisting fibrous sheaths and the pubis arrest the progress of the instrument, but the urethra may at all events become lacerated, and in fact often is so. How many times have I, when called to pass an instrument where catheterism had been unsuccessfully tried, been obliged, in order to avoid passing it again into these lacerations, to direct the point of the instrument away from the anterior wall so as to carry it backwards towards the bulb, sliding it afterwards from below upwards, so as to enter the narrow membranous portion. How many times also, finding the anterior wall of the urethra swollen from the contusion produced by the repeated pressure of the catheter against the ligament, have I been compelled to practise the *tour de maitre,* and carry the instrument along the floor of the bulb, bringing it up afterwards behind the swollen part which impeded its entrance to the narrow portion. Thus, this precept must be understood in a qualified manner, and above all should not be exaggerated; otherwise it may give rise to difficulties which would have been avoided by being less impressed with its importance."

The mucous membrane of the urethra, if the penis be not extended, may form *valvulæ* able to impede the progress of a small instrument. They are not, however, so often met with as represented by some writers who have probably confounded small bridles and cicatrices with them. They are especially found at the lower portion of the *fossa navicularis* and at the junction of the membranous and prostatic portions of the urethra. The small *lacunæ*, termed *sinuses* of *Morgagni*, are sometimes sufficiently enlarged to impede the progress of a small catheter, and are especially found in the vicinity of the bulb—the seat of the chief difficulties of catheterism. As to the *muscularity* of the entire urethra, Dr. Leroy does not agree with those who infer this from the fact of its existence in the horse, in which animal the muscles serve to withdraw the penis within its sheath.

"The urethra has no muscular fibres proper to itself, and it is quite evident to me that the spongy portion is entirely destitute of them; but, in the membranous and prostatic regions, the mucous membrane is covered by a muscular plane—a prolongation of fibres from the bladder, which, after separating to surround the prostatic portion of the canal, cover the membranous portion, and terminate in a kind of fibrous ring, which becomes confounded with the tendinous fibres of the muscles attached to the bulb, so that it might almost be said a first neck of the bladder exists immediately behind the bulb. In fact, from this point, the sensation no longer resembles that in the spongy portion; for, as soon as the end of the instrument has passed the contraction which forms the entrance of the second portion of the urethra, it arouses the desire to urine. This muscular contraction also seems to form at this orifice of the membranous portion, if not an obstacle to the exit of fluids from within, at least one to their entrance from without. Every one has observed, while injecting the urethra, that the fluid returns without passing beyond the bulb, even when a catheter has been carried as far as that; but if the eye has passed the muscular portion but a line the liquid enters the bladder, surmounting without difficulty the resistance of the true neck of the organ. But it is not only to the entrance of fluids this musculo-membranous orifice opposes itself. Instruments, when soft and flexible, have in certain cases difficulty in passing it. We are obliged to furnish them with stilettes, and keep them against the orifice, until the slight and sustained pressure has overcome the resistance. This obstacle is not only produced by the terminal ring of fibres proceeding from the bladder, but also by the contraction of those of the bulbo-cavernosi, the transversus perinei, and the external sphincter of the anus, which, coming from opposite directions to fix their attachments to the point of origin of the membranous portion, draw it in as opposite directions. It is by all these circumstances and united conditions that is produced that obstacle to the passage of instruments to which the name of *spasm of the urethra* has been given. To explain the production of this spasm, it has been supposed that, among the fibres springing from the bladder to cover the two deep-seated regions of the urethra, some are circular; but besides that, the existence of annular fibres is imaginary, the supposition of their existence is not necessary for the explanation of the phenomenon, since it is always at the orifice of the membranous region, that it is produced. There are many fibres almost annular at another point of this region, but these belong to Wilson's muscles; they are beyond the muscular plane which forms a covering to the two deep-seated regions, and spasm is not usually observed in the part embraced by them."

It must not be forgotten that the urethra varies in degree of *sensibility* in different parts of its course, otherwise a healthy may be mistaken for a diseased structure. The sensibility is acute at the meatus, the bulb, and the neck of the bladder—its degree varying exceedingly in different persons. It soon subsides, and operations can then be performed which it, at first, would seem to render impracticable. There is also great sensibility manifested at the meatus urinarius when a calculus or other cause irritates the neck of the bladder, but causes of irritation seated in other parts of the cavity of that organ exert a similar effect, and even renal calculi have produced it. "This sympathetic pain is modified according to the nature of the irritating cause. It is felt after voiding, and resembles a painful itching when there is a calculus, but is more like a burning sensation, and precedes the jet of urine in catarrh of the bladder. Most writers have noticed this sympathetic sensation, but few have observed that there is a kind of *reciprocity* between the parts, and that emollient applications to the end of the penis and local baths calm the pain of stone in the bladder. When, in lithotrity, an instrument is employed which distends the orifice too much, contractions of the bladder occur, which impede the operation and may prevent its success."

*Nature of Stricture.*

Both authors treat of two different species of Stricture, the *Organic* and the *Spasmodic*. Dr. Leroy divides the former into the following varieties, without, however, meaning to affirm that they are all to be recognised during life. 1. The *Inflammatory Stricture*. Inflammation may invade a healthy urethra, or one that is already narrowed, and when it does not terminate by resolution a stricture is the result. 2. *Fungiform* strictures depend upon a chronic vascular tumefaction of the mucous membrane, and with this most strictures commence. 3. *Valvular folds* and *bridles*. 4. Strictures which are termed *fibrous* or *callous* are formed by thickening or deep cicatrices of the mucous membrane, and engorgement of the sub-mucous tissue. From these there results an inodular substance which becomes dry, insensible, and of the pearly appearance of tendon, from which it differs in the close intercrossing of its fibres. "When a sound is passed over such strictures, one is struck with the hardness of the tissue, and with the force with which the instrument seems to adhere to it." M. Cruveilhier erroneously describes this as the only form of stricture. 5. *Turgescent Strictures* are found to exist especially in the spongy portion of the urethra, which is more easily influenced by an afflux of blood, and they give rise to several effects reputed as spasmodic. As long as the case is one of mere turgescence, relief follows the passage of a bougie, but when a chronic inflammation is developed, or breach of the mucous surface occurs, it may proceed on to the formation of a fibrous stricture, exchanging the elastic sensation it imparted to the bougie for one of a hard, horny nature. 6. *Ulcerations* are generally but secondary, resulting from the irritation caused by the urine posteriorly to the stricture; but they may be also the primary source of mischief, causing when they cicatrise one of the most unmanageable forms of stricture. 7. Although denied by many authors, *vegetations* or *carnosities*, undoubtedly do occasionally cause obstruction in the course of the urethra, especially at the fossa navicularis; and they are not uncommonly found at the orifice of the meatus in women. 8. *Varicose Strictures*. Varices of the neck of the bladder are a frequent accompaniment of an engorged state of the prostate; but a varicose state of other parts of the canal, producing stricture, has been denied by many surgeons. The profuse bleeding following sometimes even the careful use of instruments, would seem to indicate its existence. 9. *Cartilaginous Strictures* are only found in the spongy portion and seem to proceed from a changed condition of the fibrous envelope of the corpora cavernosa.

The following are some of M. Lallemand's observations upon organic stricture.

" By observing what takes place in strictures situated at the orifice of the meatus, it is easy to conceive the mode of formation of those which are deeper seated. Thus, we find inflammation at the extremity of the glans produce an increase of sensibility, swelling, and an afflux of blood, and leave as a consequence a circular induration, surrounding the orifice, and, sometimes, prolonged funnel-shaped into the interior of the canal. At other times, venereal ulcerations are formed at the meatus, and after cicatrization their edges are contracted, or their opposite surfaces unite. In both these cases, the obstacle to the flow of the urine is constituted by an *induration,* itself resulting from an inflammatory process—only in the latter there is a loss of substance besides. So of strictures deeper seated, the greater part consist but of an induration of tissues, previously softened by inflammation, but in some there has been more or less prior destruction of substance. * * * * * * * * In all the cases we have cited, inflammation has been the evident cause of organic stricture, but the phlegmasia was not confined to the villous or secretory surface of the mucous membrane of the urethra. A discharge may continue for years without causing induration, or permanent obstacle, because the products of the inflammation are discharged at the surface; while, on the other hand, inflammation of the sub-mucous tissues may cause induration without occasioning any notable increase of secretion. Thus, when a stricture follows an urethritis, it is not to be attributed to the catarrhal affection, but to the deeper inflammation which it has excited. Blenorrhagia is but an inflammation of the follicles of the urethra and prostate: by proper means, duly employed, we can soon effect its resolution. But if the inflammation has passed the mucous membrane its consequences are got rid of slowly and with difficulty; for then the materials, detained in the meshes of the inflamed tissues, are subjected, by the absorption of their more aqueous parts, to different changes. New tissues are always more dense than those at whose expense they have been produced, and the canal becomes diminished in its capacity.

" There then takes place in the substance of the urethra the same that occurs in all parts the seats of inflammation. The inflamed tissues at first lose their consistence, and are softened; but, after a time, the morbid process produces new materials, which are completely removed by absorption, when resolution takes place, but which, when this is slow or incomplete, are only deprived by the absorbents of their more fluid portions. These new elements, generally of a gelatino-albuminous nature, remain in the tissues forming diffused indurations, adhesions, more or less hard kernels, tumours, &c.—transformations constituted by a combination of the matters which have resisted absorption with the normal tissues, the whole after a while sometimes acquiring a fibro-cartilaginous consistence. * * * * * * * * * The strictures are circular, if the inflammation has invaded all the circumference of the urethra, and they form portions of a circle of different size and thickness, or present a longitudinal form, according to the seat, the extent, and the intensity of the prior phlogosis. When the inflammation is seated externally to the mucous membrane, it leaves more or less projecting tumours, which may sometimes be felt just under the skin, and narrow the canal by a direct compression."

*Spasmodic Stricture.*—Dr. Leroy believes that, under this term, authors have included different conditions, as an irritable state of the canal, the temporary turgescence of an organic stricture, the contraction of the muscles surrounding the membranous portion of the urethra, &c. Sir E. Home, believing the urethra possessed of a muscular coat, attributes this form of stricture to its partial contraction, and Brodie believes it to arise from the action of Wilson's muscles. The obstacle however occurs anteriorly to their attachments, and *spasm of the urethra*, as it should be called, rather than spasmodic stricture, is produced by the joint action of the bulbo-cavernosi and other accessory muscles of the urethra, and of the species of muscular ring formed at the commencement of the membranous portion of the urethra by the muscular plane which proceeds from the bladder. In confirmation of the efficiency of this last, the author observes that he has almost always observed spasm of the urethra at the commencement of hypertrophy of, and in irritation of, the prostate gland, which induce contraction of this muscular part. The obstacle to the passage of instruments thus produced, requires gentle pressure made by not too small an instrument. A gum-catheter, containing an iron stilette, is to be placed in contact with the muscular orifice until this opens, and when the point of the instrument has entered even but four or five millimetres, the stilette may be withdrawn, as the catheter then passes easily enough. Sometimes the obstacle is so great, that the attempt to pass the instrument must be postponed. Fortunately this muscular constriction does not produce retention of urine, and in most cases we can wait until it subsides. When retention, caused by affection of the neck of the bladder itself, exists, this first obstacle must then at once be overcome, although even then the urine will not flow until the catheter has actually passed the neck of the bladder —the true seat of retention, complicated in the case now supposed by spasm of the urethra. This spasm may also be combined with organic stricture, as shewn by the grasping of the bougie by this muscular orifice. It is sometimes present in stone of the bladder, disappearing after the removal of the calculus. It also is occasionally produced by rheumatism, but much oftener by an engorged prostate. Compression of bougies by the urethra is no proof of spasmodic stricture, since it may be produced by mere vascular distension of the canal, induced by the irritation of the instrument.

"There is then really no such a thing as a spasmodic stricture, or at least this term cannot be properly bestowed upon any of the obstacles to which it has been applied; for in one case there is muscular contraction, or spasm, but no stricture, or obstacle to micturition in the spot where the catheter meets with resistance; and in the other case, where there is really stricture, the sudden variations in the diameter of the contraction proceed from some sudden inflammation or temporary turgescence, or a muscular constriction, which would be of no avail in the production of retention, if an organic change of structure did not already exist."

M. Lallemand, however, and we think with entire reason, acknowledges the occasional existence of spasmodic stricture properly so called. He has observed several cases, in which the impressions given to the wax bougie, passed to examine the state of the urethra, have exactly resembled those produced by organic stricture ; and he has frequently only become alive to the true nature of the obstacle, by observing it at different examinations to occupy different portions of the canal, being capable indeed of being produced extemporaneously in any part of it. In other cases, voluminous instruments pass with greater facility than smaller ones. Such spasmodic contractions are intermittent, but, during their existence, forcible attempts to overcome them irritate the urethra, and add to their constrictive power.

"What leaves no doubt as to the spasmodic nature of these strictures is the difficulty there sometimes exists in withdrawing an instrument after it has penetrated the urethra. It is so closely grasped that the penis is raised by every attempt to remove it; and sometimes it is necessary to leave it a half or a whole hour before it can be extracted without causing new contractions.

"There exist then temporary constrictions of the canal simulating permanent organic strictures, although the urethra may be the seat of no induration or cicatrix, and such coarctations can only result from spasm. Dysury and retention arising from them must not be confounded with these states produced frequently by an inflamed prostate. In spasm, retention is sudden, changeable, and the urine is passed in a full stream during the intervals. In inflamed prostate, the dysuria increases gradually to complete retention, persists long without intermission, and, when it yields to antiphlogistics, never does so suddenly. Besides, the patients feel an unsupportable heaviness about the perineum and rectum, and frequent desires to go to stool. The prostate, examined by the rectum, is found large and painful, and a large catheter passes without obstacle into the bladder."

### *Causes of Stricture.*

Whatever causes inflammation in the urethra may produce stricture. *Gonorrhœa* is a more frequent cause than any other, but for its operation some original disposition seems necessary, or the affection would be infinitely more common. A first gonorrhœa frequently only encreases the susceptibility of the urethra, the stricture rapidly following a second attack of the disease. Dr. Leroy does not believe the disease is produced in consequence of the use of *injections,* and he has notes of 107 cases in which it came on without any injection having ever been used; and where these have been employed, how seldom have they ever reached the membranous portion of the urethra, where nine-tenths of the strictures are located. Injections should rather seem a preventive by removing the obstinate discharges which tend to end in ulceration. Dr. L. does not however mean to state that injections are always innocent, and has known a great number of cases in which stricture has followed their employment at the commencement of the disease, or when used too strong. He regards the *nitrate of silver* as especially giving rise to stricture, as well as to phlegmasia of the prostate and cervix vesicæ. M. Lallemand also regards gonorrhœa as the most frequent cause of stricture; but, in a few instances, has known it to follow masturbation, or venereal excesses, even unpreceded by any running.

Next to gonorrhœa, external or internal violence inflicted on the urethra is the most frequent cause of stricture, such as contusions or lacerations of the perineum, (M. Lallemand states that strictures are found more frequently in the English than the French, in consequence of the passion for horse-exercise prevailing among the former), violent or injudicious catheterism, &c. It not unfrequently results in the lower orders of France from their inflicting a violent blow upon the urethra, in order to what they term *break the cord* in the chordee of gonorrhœa. Dr. Leroy says he has met with but about fifteen cases, in which the stricture was unpreceded by sexual intercourse, discharge, violence or other cause of inflammation. He truly adds, that few are the patients whose statements on this head can be relied upon, "for there are many who have had discharges and obstinately deny it, adding, at the same time, they have no motives for concealing the truth."

Persons who in childhood suffered obstinately from enuresis, or in youth urined with difficulty or too frequently, especially those of a lymphatic or scrofulous constitution, seem much predisposed to stricture; and, in them, it often takes on some unusual form, as a fungiform or hæmorrhagic, state of the neck of the bladder, spasm of the urethra, &c.

*Seat of Stricture.*

Dr. Leroy observes that, as authors vary so much in their statements of the natural length of the urethra, so they do as to the precise distance of the strictured part—according to his own observations on the living subject—

"1. Nineteen-twentieths of strictures exist at a depth varying from 5 to 6 inches, (M. Lallemand states it at from 16 to 20 centimetres), *i. e.* immediately behind the bulb, at the commencement of the membranous portion, beneath the pubis, where the urethra is naturally narrower. 2. Next in frequency are strictures of the posterior lip of the *fossa navicularis*. 3. Those of the meatus. 4. Strictures of the spongy portion, situated 2 or 2½ inches from the meatus, at the root of the penis, at the point where the canal is naturally somewhat narrow, and where, in a state of flaccidity, the penis is bent upon itself. 5. Various authors have met with stricture of the prostatic portion, and Lallemand has found it extending to the neck of the bladder."

Strictures are frequently multiple, and M. Leroy believes that, in nearly one-half the cases, there is a second 7 or 8 millimeters from the first. He has met with eleven and Lallemand with seven in the same urethra. When two strictures are seated at a considerable distance from each other, the intermediate space is often dilated; but, when they are very near to each other, their bases meet, and this portion of the canal is then smaller than in the natural state.

*Number.*

"When there is but one stricture, providing it is not too near the neck of the bladder, the urine is projected to a certain distance, although the stream may be very small, bifurcated or twisted. When there are several, the urine falls perpendicularly between the patient's feet, and when the number is great, or the narrowness extreme, the desire of emitting increases in frequency, because the bladder is only incompletely emptied. Its contractions cease, before it has entirely expelled its contents, and when this condition persists, the reservoir becoming more and more full, the emissions succeed each other so frequently as to constitute a species of incontinence of urine.

"When there are several strictures, one of these, generally the oldest, is always narrower than the others. If there are others between this and the bladder, they are larger than those placed between it and the meatus, because the urine, habitually retained by the chief obstacle, acts upon and distends such as are situated posteriorly: or they are indurations which only occupy part of the circumference of the urethra, and do not prevent the distension of this portion of the canal. The anterior portion uninfluenced by the jet has the greater tendency to become narrower. In general, the difficulty of treatment is proportionate to the number of strictures, not only because catheterism is more difficult, but because nothing has been done for the patient while but one existed."—*Lallemand*, p. 131.

*Length.*

Although, in the majority of instances, strictures are very short, yet most authors speak of having occasionally met them from one to two inches long, and Dr. Leroy gives the figure of one which extended from within an inch of the meatus to the bulb, *i. e.* nearly five inches. It is especially in the spongy portion such long strictures are met with, but examples are adduced of the whole, or nearly the whole, of the membranous portion being contracted. Several reputed long strictures however are really numerous small ones, situated very near to each other, and the ordinary means of exploration has often led to the belief of the stricture being longer than it really is.

"There are some of such slight extent that they resemble a kind of diaphragm, and are passed as soon as the instrument enters their orifice; others, again, are 25 millimetres, and more, in length. The opening of the first is usually irregular or unilateral, and the catheter arrives at the opposite extremity as soon as it has entered the stricture. The orifice of long strictures is usually regular and funnel-shaped, because the inflammation has occupied the whole circumference of the canal. The bougie enters easily, but meets with resistance and friction over a great extent, as its extremity may easily hitch against some points of the surface in consequence of the irregularity of this, or the axis of the obstacle not being exactly followed. This stricture then is easily entered, but quitted with more difficulty. It will be readily understood that there is every intermediate size between the diaphragmatic and the long stricture. All other circumstances being alike, the disease is difficult of cure in proportion to its extent."—*Lallemand,* p. 128.

*Sensibility.*

M. Lallemand observes that this becomes diminished in proportion to the age of the stricture, and that, when an excessive sensibility is manifested, it does not proceed from the indurated portion, but from the irritated or inflamed condition of the neighbouring parts produced by the incessant attempts to urine. Sometimes, however, the mucous membrane lining the obstacle becomes excoriated or even ulcerated, and then excessive pain is felt in attempting to pass an instrument, and it is to such cases authors refer when they speak of the sensibility of strictures, and the immediate effects of cauterization.

*Effects.*

Although the portion of the urethra *anterior* to the stricture retains for a longer or shorter period its normal calibre, yet, after some years, or under the influence of certain causes, it becomes narrowed. M. Leroy believes this to be produced by a chronic state of inflammation, induced in a great measure by the irritating properties of the altered urine. It is rare for this narrowing to be uniform, it commonly presenting itself in the form of a succession of little indurated points. If caustic be applied to the urethra under these circumstances, these prominences disappear, because this substance, by its irritation, produces tumefaction of the intermediate portions, so that the entire canal becomes equably narrowed.

Sometimes, however, the urethra is found *dilated* immediately anterior to the stricture. This is especially the case when patients have been in the habit of passing instruments themselves, without reaching the bladder.

By this means the natural excavation of the bulb is enlarged, and this part is not infrequently perforated. In some persons this anterior dilatation results from an anormal laxity of the spongy portion, often induced by the traction of the urethra by the patient during dysuria. Many persons produce this dilatation also by another practice, viz. that of endeavouring to augment the size of the small jet of urine by occasionally interrupting its course by compression opposite the fossa navicularis, ignorantly believing that, in this way, they were dilating the stricture. Sometimes the relaxation of tissues so produced is not uniform, and a kind of cells are produced in the urethra.

*Posteriorly to the stricture* the canal is dilated, and various changes occur in the prostatic portion of the urethra.

"In almost all subjects affected with considerable stricture upon whom autopsies have been performed, this is found injected, thickened and fungiform. It is in this sense only we understand what are called fungosities or carnosities of the urethra. The follicles of the prostate sometimes become elongated to 15 millimetres, and admit a probe as large as a crow-quill—the prostate itself being changed in appearance and increased in size. * * * In proportion as the prostate is irritated the functions of the rectum become interfered with, a feeling of weight at the anus, and illusory desire for stool being perceived. The urine becomes ropy and viscous, and leaves a deposit of long, glairy, elastic filaments, resembling a trembling jelly, adhering firmly to the utensil, and becoming much elongated before they are detached from it. This glairy deposit arises from the increased secretion of the prostate. * * * * * Sometimes even the prostate becomes destroyed by suppuration; it can no longer be felt from the rectum, or only so in part, the remainder being soft and flattened, and false routes are then easily made, especially by the small instruments the narrowness of the stricture may require.

"It may be easily imagined that this irritation of the mucous membrane is propagated to the ejaculatory canals, the vesiculæ seminales, &c., and the frequent occurrence of swelling of the testis as a consequence of stricture proves this to be the case. It results from this state of irritation of the reservoirs of the semen and its excretory canals, that coition is promptly followed by emission, the patients are often subjected to nocturnal pollutions, and in the one and other case the pleasurable sensation is often accompanied by one of a painful character. Later, when the irritation is increased, emission takes place in a state of semi-erection, and without any sensation indicative to the patient of the frequency of its occurrence during the expulsion of the urine and fæces. Then the appetite becomes lost, and the digestion difficult; the patient gets thinner and weaker, and falls into a state of apathy or hypochondriasis."—*Lallemand.*

M. Leroy observes that it requires generally several years to have passed before such disorder of the generative functions is produced. Contrary to what would seem from the turgescence *à priori* to be the case, the emission of urine is usually more easy after coition.

The muscular coats of the bladder become much augmented in size, the mucous membrane projecting as herniæ between the bundles of fibres. The urine, by reason of the absorption of its watery parts, becomes a source of irritation to the organ, and gives rise to the secretion of abundant mucosities, which, by their decomposition, give the fluid a fetid smell. M. Lallemand observes that the deposit these form is always, whatever its appearance, mobile, and thus to be distinguished from the glairy adhesive secretions of the prostate. In the course of time the ureters and pelves of the kidneys become dilated, and in one case M. Lallemand found the former so large as to be at first mistaken for portions of the small intestine. The kidneys become enlarged, soft, pale and spongy, and at last inflamed, abscesses forming in their substance in various numbers, and at different epochs.

"It is almost always from the kidneys that the *pus* found in urine has proceeded, as M. Lallemand has often had occasion to shew his pupils at the autopsies. It is seldom furnished by abscess of the prostate, and it is doubtful whether the surface of the bladder ever secretes true pus. The attentive examination of the urine in stricture is far too much neglected. In general, when it is only turbid, without deposit or cloud, there is only irritation of the mucous surfaces. If there is a flocculent cloud which does not reach the bottom, we must suspect diurnal pollutions. If there is a thick, mucous, or puriform moveable sediment, a catarrhal inflammation of the bladder exists; while, if the deposit is glairy, elastic, and adhesive, it proceeds from the inflamed follicles of the prostate. If the deposit is purulent, and the prostate small, flat, and of difficult recognition, this has been destroyed by suppuration; but when the prostate is healthy it is most probable the pus proceeds from the kidneys."

We need not follow M. Leroy in his description of the rational symptoms of stricture, but at once proceed to his observations upon its

*Exploration.*

After describing the bougies contrived by Arnott and Ducamp for exploring the urethra, and shewing their insufficiency in bad cases, and their uncertainty in others, M. Leroy states he finds a modification of Charles Bell's urethral probe forms the most effectual instrument. This consists in a flexible bougie terminated by a small round or olivary ball, and varying in size according to the degree of stricture. This little ball facilitates the passage of a small instrument over the folds of the urethra, and lessens the probability of its detention at the commencement of the membranous portion. The instrument is passed to the neck of the bladder, and then gently withdrawn without exerting traction upon the penis; the number and length of the obstacles which oppose its withdrawal are noted upon a scale marked upon the shaft of the bougie. Although M. L. believes the wax bougie alone is a most uncertain and imperfect exploratory instrument, he uses it as a means of verifying the results already arrived at by means of the other, and it is indispensible when the existence of vegetations, fungosities, or gravel in the urethra, is suspected. So, too, the impressions produced by Ducamp's instrument, are of great use in indicating the form, size, &c. of commencing false passage. When the stricture is excessively small, its exact size and nature cannot be explored until, by the use of the smallest gum-bougies, we have produced a sufficient degree of enlargement to allow the ball to pass.

"In spite of the blind confidence of those who admit of no impassable obstacles, and who state that, wherever the urine passes, catheters or bougies ought to find a passage, it is no less true that there are strictures which admit no instrument, whatever may be its tenuity, and yet allow the urine to filter through. This is probably less owing to their closeness than to their form. This kind of obstacle is sometimes produced by projections or thickenings existing alternately on the two sides of the canal, so that it acquires a zigzag direction; and that this is the case is shewn by the fact, that frequently, by twisting the point of a very fine bougie into an irregularly spiral form, it adapts itself to the sinuosities of the canal, and passes without difficulty."

M. Lallemand employs an instrument in no-wise superior in its advantages to a wax bougie, and far more dangerous, inasmuch as the composition is sometimes detached from the catheter, and left in the obstacle, producing retention of urine. He takes a large gum-catheter, and having cut it across above the eye, scrapes some of the composition from near its extremity so as to have some of the threads of its framework hanging loose. These are dipped in a mixture of pitch and wax of a sufficient consistency to allow of the moulding by the fingers of a narrow stalk, which in this way form's a portion of the instrument. Into the catheter he passes an elastic gum bougie exactly fitting it, which is less rigid than a metal stilette would be, and possesses sufficient elasticity, while it is strong enough to cause the wax to penetrate the obstacle. Carried down to the stricture, after a-while the wax becomes warm and soft, and, on pressure being made, a portion of it penetrates the obstacle—the elastic body supporting it perpendicularly in the axis of the urethra.

Dr. Leroy has a section upon the *differential* diagnosis—*i. e.* upon the means of distinguishing the various species of stricture he has described. The nature of the stream of urine does not indicate this, or even for certain the existence of a stricture at all. Old strictures, as a general rule, pass into the *fibrous* or *callous* state ; but not necessarily so, for in the present work cases are cited wherein the stricture, even at the end of 20 or 30 years, retained its vascular character, and yielded to simple dilatation in a few days. If a bougie is grasped tighter and tighter by a stricture of the spongy portion for half an hour, and the constriction, then relaxing, is reproduced upon moving the bougie, the stricture is probably an *erectile* or *turgescent* one. When, after having been strongly grasped, the bougie soon becomes mobile and remains so, the stricture is fungiform, being produced by vascular distension. If a very small bougie has been tightly constricted and loosened as it proceeds on, and especially if it passes better when its point is twisted, a stricture with a succession of projections is present. The fungiform and vascular strictures again are sensitive and irritable, while the fibrous is callous, but ulceration of the mucous membrane produces irritability ; and the membrane posterior to any stricture is often very sensitive, so that the excitement of pain after passing a stricture is by no means, as stated by some, a sign of false passage having been made. Abundant bleeding, especially in jets, is a sign of a varicose stricture. When we find a medium-sized catheter arrested, and a conical gum bougie pass easily, we may admit the presence of a *valvular* stricture, which is more common than is believed. When the valve is inclined towards the bladder it may produce retention, and yet admit the passage of a large bougie. At the best these marks of diagnosis are but approximative.

*Treatment.*

Before proceeding to discuss the treatment of stricture, properly so called, this author makes some remarks upon *contraction of the prepuce and of the orifice of the urethra.* A chronic phymosis gives rise to many local inconveniences, but may also lead, by the obstacle it presents to the free passage of the urine, to the most serious affections of the deeper-seated parts, as inflammation of the urethra, bladder, testes, &c. In a case related, the urethra from this cause was destroyed to the extent of 4 centimetres. On the other hand, the prepuce itself may become inflamed and changed in structure from the contact of unhealthy urine. Incision or removal of the prepuce, according to the nature of the case, is required. The orifice of the urethra itself is not unfrequently closed by a valve or bridle; and in some persons it is anormally small. This last state may cause, during the whole of life, little or no inconvenience; but, if disease of the bladder or other parts of the urethra occur, it will require incision and dilatation. This part is also very prone to become converted by inflammation into a fibrous inodular tissue, and the state is only aggravated by the employment of dilatation by large catheters, while smaller suffice to empty the bladder and allow the inflammation to subside. Caustic, which has been frequently used in these cases, much aggravates the malady. Incision, which is usually required, causes considerable pain and hæmorrhage (except where the obstacle is only valvular), and the best means of restraining the latter, is to introduce a large catheter, and retain it there as long as its compression is required.

*Treatment of the Varieties of Stricture.*—Dr. Leroy, like all other prudent practitioners, advocates no exclusive method of treating stricture. Indeed, he excludes no mode whatever, believing each is adapted to some particular form of the disease. His ingenuity in devising modifications of instruments, in order to meet every possible emergency, is indeed inexhaustible, and we are certain that many of those depicted in these pages are superfluous; while it would certainly require a life-time to acquire the nicety of touch requisite for the employment of some of them. Nevertheless, the author's precepts are sound and useful, and happily may be carried into effect with a less complex "arsenal." He places strictures, as regards treatment, in three categories. 1st. Those which allow both urine and bougies to pass. 2nd. Such as allow urine but not the finest bougies to traverse them, and—3rd. Such as present complete obstacle to both.

1. *Strictures which allow the passage of both the Urine and Bougies.*—A mere *valvular* stricture will frequently be destroyed, never to re-appear, by a single passage of a good-sized instrument, and when the concavity of the valve is directed backward, the ball-bougie, before mentioned, becomes, as it is withdrawn, an effectual instrument, both for its detection and removal. A stricture too in its earliest stage, when there is but slight thickening of the mucous membrane, is sometimes cured by a single catheterism, especially if this ball-bougie be drawn to and fro over the projecting portion. Valvular strictures with a large base, or possessed of much elasticity, resist these means and require incision.

*Dilatation.*—This is performed in three different manners.

"The catheter may be left in the urethra, and changed but every three or four days—the slow or *permanent dilatation:* it may be changed every 6 or 7 hours, which forms the continuous, sudden, or to use a favorite term in medical language, the *coup-sur coup dilatation:* Lastly, dilatation may be obtained by the retention of the bougie for a period varying from 5 minutes to 1 hour, gradually increasing their calibre in a progressive manner very important to observe, which is the *temporary* or *progressive* dilatation. Each of these methods is proper for certain strictures; but it is seldom that we can at once determine which mode is likely to best succeed. We must, therefore, commence with the most simple and the most convenient to the patient, and, in these points of view, *temporary* dilatation is to be preferred. It does not oblige the patient to abandon his business, or even his pleasures, during treatment, requiring only half an hour's repose daily. It is not painful, and often cures as radically and as promptly as the permanent dilatation."

M. Leroy prefers, for the generality of cases, to all others, the *gum-elastic bougies*, by reason of their simpleness, resistance, and polish, employing the capillary, spiral, conical, or curved one, according to the exigencies of the case.

*Temporary Dilatation.*—When the point of the bougie becomes grasped by the stricture, if it is left for ten minutes or so, it will often be found that it can then be pushed in with a very moderate force; and attempts may be continued to pass a stricture as long as they occasion neither pain, fatigue, or bleeding, but when these are produced, any further efforts should be deferred until next day. When the stricture is passed the bougie may be retained for a period varying from a minute to half an hour, according to the sensibility of the urethra. The majority of patients support the bougie for a quarter, half an hour, or a much longer period if required, without the least inconvenience; but some persons manifest the greatest irritability unless it is very shortly withdrawn. After two or three applications this sensitiveness usually subsides, but in some rare cases persists. Bleeding, belladonna, opium, &c. are of little use in relieving it. The best plan is not at first to attempt any dilatation, but to begin by accustoming the urethra to the presence of a smaller instrument than would be required for that purpose.

"An invariable rule in temporary dilatation is never to employ force in passing the bougie, introducing one at first which passes freely or enters with but slight effort; and never to increase the size of the bougie from day to day, *but at the same sitting, i. e.* commencing with that which passed freely the day before. * * * * In the majority of cases the diameter of the bougie may be increased half a millimetre each *seance*, which makes about 20 days as the ordinary term of treatment. The following are the steps I usually pursue. In the two first *seances* one bougie only is introduced and left in a quarter of an hour; in the third, the bougie of the day before is first introduced, and then, after a quarter of an hour, one half a millimetre larger is employed, which in its turn is retained a quarter or half an hour: on the fourth, after the two first bougies have been passed, a third and larger is tried, and so on, increasing half a millimetre daily—taking care to commence by the bougies which entered easily the day before, causing each to be retained from five to ten minutes. Every second, or when the stricture is callous, every third day, the smallest of the series is discontinued, commencing with the second, then with the third, until the largest, of 7½ or 8 millimetres, passes through at once. When four millimetres are reached, curved bougies pass with less violence than straight ones. * * * * In the great majority of cases the painful sensation following the augmentation of the size of the bougie is relieved in ten or fifteen minutes, and the instrument grasped tightly at first then becomes free in the canal; so that if we did not allow it to remain in longer than five or ten minutes, as advised by some, and if we do not wait for the relaxation which follows the excitement produced by the introduction, we delay the treatment, for it is only after such relaxation the bougie can exert any mechanical effect. The *seances* usually last from 30 to 40 minutes, during which two or even three bougies are introduced, and retained from 10 to 15 minutes each. There is infinitely less irritation produced by using two or three bougies at a sitting than by employing at each a larger one at first than had ever yet been tried. * * * * * * This treatment by temporary dilatation, which continues from 10 to 30 days, very often produces, notwithstanding this is denied, durable cures: and does so almost constantly when the stricture is fungiform, vascular, or produced by a thickened mucous membrane. I have had occasion to see patients whom I thus treated in 1826–8, that is more than 15 years ago, and the stream of urine in these cases has continued the same size as immediately after treatment, *without a single bougie having been introduced since that time.*"—*Leroy*, p. 209–12.

Temporary dilatation does not give rise ordinarily to urethritis or any discharge, and much oftener suppresses such if it exist. In some irritable subjects, however, a single application will produce this. The testicle also occasionally becomes inflamed, when the treatment of the stricture must be suspended, but may be resumed *before resolution has completely taken place,* as soon as the more urgent symptoms have disappeared. The orchitis seizes the sound testicle in preference to re-appearing in that in which it has once appeared. To prevent this accident, a suspensory should be worn during treatment.

M. Leroy relates many cases in which dilatation, otherwise impossible, became practicable by means of the small bougie with spiral end.

*Rapid, or Coup sur Coup, Dilatation.*—M. Leroy observes that this is required, when the stricture, after a certain degree of improvement, remains stationary, which is the case in the fibrous variety. It is highly useful also where rapidity of treatment is the main point, as when the patient's engagements allow of his devoting but a short space of time to himself, or when calculus of the bladder complicates the stricture. By this mode of treatment the catheter is increased in size every eight or ten hours, and the time occupied is but four or five days. This is the plan M. Lallemand now prefers, having abandoned his former predilection for the employment of caustic.

"Prolonged dilatation, even yet so generally practised, was abandoned by M. Lallemand, in consequence of its injurious effects; but more lately, having observed that cauterization is not applicable to all cases and does not prevent relapse, he sought some new method, and proceeding by progressive steps, he was induced to adopt the plan of *dilating* the stricture *rapidly*, having observed how easily we may pass from one sized instrument to another, and above all having proved that the cure is as durable as that produced by any other treatment; so that he was enabled to produce in two or three days results which formerly he could not have hoped for under as many months—preserving also all the advantages of dilatation without its inconveniencies."

M. Leroy remarks that, when a regular increase of their size is observed, there is seldom any difficulty in passing the instruments, the first indeed being that which least easily enters. He employs the elastic gum catheter without the stilette, unless there is spasm of the urethra when this is required, in order that some resistance may be offered. He insists upon three rules being observed. 1. To wait before passing the second catheter until the irritation and swelling produced by the first subside, which they usually do in 24 or 36 hours. If an increase of the size be attempted before this, success is prevented; while, if the passage of the second be delayed, the others may rapidly follow. 2. Suspend the treatment when it produces painful micturition, bloody urine, &c. These symptoms rarely appear before the fifth day, when the treatment is in fact concluded, and they disappear on the removal of the instrument. 3. After a bougie of $7\frac{1}{2}$ or 8 millimetres has been passed and retained in the urethra for 24 hours, some days rest must be given the patient; and then, to assure the treatment, the three last numbers should be passed for half an hour during a week or so.

*Permanent or Slow Dilatation.*—In this mode the catheter is changed only every fourth, fifth, or eighth day. Although it is still much employed in the French Hospitals, M. Leroy considers there are few cases of even hard and fibrous stricture that require it, and, to be permanently useful, it must be followed for some days by temporary dilatation by means of large instruments, or retraction of the tissues occurs. The prolonged retention of the catheter is of indubitable advantage in cases of retention of urine from enlarged prostate, as it often produces a diminution of the engorged tissue. The bad effects which sometimes arise from this permanent dilatation have been strongly stated by Lallemand. Among these may be mentioned irritation of the mucous membrane and obstinate chronic catarrh of the bladder, inflammation, softening, and even perforation of that organ, the breaking or incrustation of the catheter, obstinate prostatic enlargements and discharges, swelling of the testes, deep-seated inflammation of the urethra, &c.

*Flexible Catheters with Short Curves.*—Dr. Leroy speaks highly of the great benefits which have resulted from the employment of gum-elastic catheters with a curve of but 34 millimetres. They are especially useful when there are deviations from the natural course of the canal, especially when these are produced by enlargements of the prostate.

*Cauterization.*—Dr. Leroy criticizes those practitioners who have frequent recourse to this, especially in strictures of the *spongy* portion of the urethra. He believes, also, that the *porte-caustiques* in use act upon too large a portion of the urethra, implicating the sound structures, since strictures as a general rule are very short. He has invented another, which consists of a thin *porte-caustique* having a knobbed or olivary extremity and apertures at its sides; when this has been passed through the stricture, a narrow stilette containing the caustic is passed into the tube and the caustic carefully applied at the side as the instrument is withdrawn, thus forming the *retrograde cauterization*, applicable only where small instruments can pass the stricture, but for which dilatation has proved insufficient. He never uses this until dilatation has been tried. He dwells upon the great necessity of caution in the employment of caustic, for if it be applied too deeply or too frequently, additional inflammation, instead of interstitial modification, is excited, and the stricture becomes harder than ever. A few days after each cauterization, the state of the stricture should be examined by a bougie, and, if found soft and yielding, the treatment should be completed by dilatation.

M. Lallemand, formerly one of the chief advocates for the use of caustic,

"After having rendered its application more simple, easy, and safe, has nevertheless renounced its employment in most cases. The first objection to cauterizing the interior of strictures, is the difficulty of limiting the action of the nitrate of silver to the portion of the canal where the obstacle is situated, so that the healthy parts anteriorly or posteriorly may not become affected by it. The next is the tendency to relapse after a longer or shorter period, and the necessity at last of having recourse to dilatation in patients who were regarded as cured by caustic. It does not follow that this method should be entirely rejected, for there are cases in which it cannot be replaced. Thus, when the interior of a stricture is excoriated or ulcerated, or the neighbouring mucous membrane is sensitive or inflamed, the presence of a catheter produces spasms and nervous symptoms which prevent the patient supporting it, and are renewed by any fresh attempts at its introduction. Here cauterization changes the vitality of the parts, leads to the cure of complications, and causes the disappearance of the excessive sensibility."

*Scarification.*—This is recognised by all as a useful proceeding in the case of bridles, valvular folds, &c. but difference of opinion exists as to its applicability to stricture which resists dilatation and caustic : but M. Leroy has no doubt of its occasional utility, conjoined with dilatation, in the treatment of callous stricture, although it often fails. In other cases, without curing stricture, it has rendered the passage of bougies more easy.

2. *Treatment of Strictures which allow the Urine but not Bougies to pass.* —In these cases there is not retention of urine, and they are to be treated by either the continuous pressure of a bougie or direct cauterization. M. Leroy observes, that the constant retention of an instrument in close contact with the strictured part is difficult, but when it can be accomplished, and he proposes an apparatus for the purpose, the stricture often becomes softened and tractable. Another plan, which does not require confinement in bed, is to press the end of the instrument against the obstacle for a quarter or half an hour daily. After each sitting, a small bougie should be attempted to be passed. This plan wants patience, for whole weeks may pass without any apparent progress being made. When a stricture cannot be passed, *direct cauterization* becomes also a proper mode of treatment, the retrograde being that which is to be put in force in cases where small instruments can be introduced. The application of the nitrate of silver requires, however, the greatest nicety to avoid getting out of the tract of the urethra.

3. *Treatment of Strictures producing complete Retention of Urine.*—Here unnecessary delay endangers life itself; yet, before resorting to more forcible measures, the various descriptions of bougie calculated to pass difficult strictures must be tried, in conjunction with the employment of bleeding, baths, &c. Dr. Leroy has derived no benefit from the application of belladonna in these cases, but he has known tobacco-smoke produce a favourable relaxation. If other means are unavailable, *Forced Catheter-*

*ism* must be had recourse to, but the thrusting of large metal instruments through the obstacle, as recommended by Mayer of Lausanne, is a very dangerous practice, tending as it does to produce lacerations. A small catheter is to be pressed rather than forced against the obstacle for the space of an hour. During the first quarter of an hour the pressure is sometimes more advantageously made by means of a conical catheter, substituting a cylindrical one when its point has commenced the penetration. M. Leroy recommends *incision* of the urethra posterior to the obstacle, only in case of a calculus being there detained, in which case he makes it through the rectum. During 20 years' practice he has had only once to *puncture the bladder,* and recommends that this should also be accomplished through the rectum.

*External Incision of Strictures.*—M. Leroy, we have seen, in cases of impassable stricture, occasionally opens the urethra posteriorly to the stricture; but M. Lallemand not infrequently cuts down upon the strictured part itself. He does this when the obstacle to the passage of the urine is situated externally to the mucous membrane, and is constituted by tumors or indurations developed in the spongy tissue, or more superficially. Such nodosities may be found near any part of the canal, and dilatation of the urethra only temporarily repels them, while caustic applied to the urethra is of course hurtful. M. L. finds, by cutting completely through these obstacles, he induces their suppuration (which sometimes occurs naturally), and eventual removal. The canal itself must be penetrated, if this is necessary to secure a complete division of the nodosity.

M. Lallemand practises the same operation under other circumstances. Thus, in a very old case, the stricture was not excessively narrow, but the urine passed only guttatim, and the urethra was so irritable as to bear no instrument whatever to pass, an incision was practised with the best effect. In another case, the stricture consisted of an elastic ring, like caoutchouc, which always resumed its former contracted state the instant the catheter was withdrawn.

"In every case of retention of urine produced by an obstacle accessible to a cutting instrument, it is better to have recourse to incision than to forced catheterism or puncture of the bladder. Guided by a large instrument passed as far as the obstacle, this must be exposed by a free incision of the skin, extending above and below it, so that a clear view may be obtained, and danger of infiltration of urine prevented. The portion of the urethra opposite the stricture is next to be divided, so that its anterior orifice may be brought into view, or that it may be found by a small grooved director passed in different directions, along which a narrow bistoury is to be slided, and the whole induration divided. If, as is very improbable, this anterior orifice cannot be found, the portion of the canal immediately posterior to the stricture now distended by the urine must be opened, in which case the obstacle would be penetrated from below upwards, and from behind forwards. The lips of the wound are to be brought together over a catheter, which is to be left in the urethra until cicatrization occurs. This manner of proceeding offers great immediate and consecutive advantages over forced catheterism and puncture of the bladder. It is less dangerous, is a more certain remedy for the retention, and procures a durable cure. * * * * * * It may be objected, the stricture may be such as to prevent our penetrating the obstacle from before or behind; but, even in this case, the obstacle would not be more easily overcome after puncture of the bladder. The worst that could happen after the incision would be the formation of an urinary fistula behind the stricture, an inconvenience surely far less than that which would be occasioned by the retention of a catheter in the bladder above the pubes."

*Traumatic Strictures.*—M. Lallemand makes several observations upon traumatic stricture, in which the urethra becomes narrowed in consequence of external violence, rupture, loss of substance, unskilful catheterism, laceration by particles of calculi, ulcerations, &c. The *treatment* must depend upon the nature of the cause, and the period when the case is seen. After external violence, if an antiphlogistic treatment be vigorously pursued, complete resolution may sometimes be obtained. When retention of urine occurs, a small pointed catheter would be likely to induce false passage, and a very large one to distend the parts too much, so that a middle size should be passed. If the *hæmorrhage* from the canal is moderate it is highly useful in depleting the parts; but, if it become too profuse, it is best arrested by leaving as large a gum-elastic catheter in the urethra as can be passed, which at once provides the requisite compression and guards the parts from the contact of the urine. As soon as there is no fear of augmenting the inflammation by the presence of a foreign body, larger and larger catheters must be introduced in cases of traumatic stricture, so as to keep the canal distended during cicatrization. The cure is somewhat delayed in this way, because, by separating the edges of the wound, there is a larger space to fill up, but the cicatrix so produced, by being thinner, possesses less contractile power and is more easily dilated subsequently. If, after the treatment has ceased, we find the stream of urine become smaller, we must have recourse again to the catheter, beginning with a small size. The probability of this subsequent dilatation being occasionally required is not always sufficiently impressed upon patients. The older the traumatic stricture is, the more perseveringly must dilatation be repeated or prolonged ; for, without this, the contractile tendency of the cicatrix will never be overcome. As catheterism becomes easier, the patient should be taught to practise it on himself, as in this way the case will be more perseveringly treated. *Cauterization* is not proper in these cases, except when employed very lightly in the thin membraniform strictures which result from ulcerations; but in all cases dilatation is also eventually required.

*Spasmodic Stricture.*—M. Lallemand observes of this—

" Its treatment requires neither permanent dilatation or cauterization, although a light superficial application of caustic has in certain cases advantageously modified the mode of action of the mucous membrane. Narcotics, employed internally or externally, are usually preferable. Narcotic injections, thrown into the canal and kept there by pressure on the glans, or the passage of a catheter smeared with opiated ointments down to the obstacle, occasion a local torpor which allows the instrument to be gradually carried into the bladder. The action of these substances is not always confined to the canal. A patient suffered from fever and alarming nervous symptoms every time that catheterism was attempted. Morphine ointment was employed, and the absorption was such, that symptoms of poisoning by opium manifested themselves. As soon as they were dissipated the instrument passed freely. These patients require that catheterism should be performed with the utmost gentleness and patience. It is not enough to warm the catheter and smear it with opiates, the surgeon must also suspend all pressure when he finds it grasped or stopped, and quietly wait for the subsidence of the spasm before endeavouring to advance a little farther. Without such circumspection he does much mischief, although the catheter be not too small or pointed; and certainly he would not reach the bladder, for the slightest error increases the constriction of the canal, any laceration would render catheterism impracticable."

Dr. Leroy's concluding Chapter treats of the important subject of the

*Complications of Stricture.*

1. *Calculi in the Urethra.*—Some persons pass small calculi in truly surprising numbers, when the urethra is free; but, when this is not the case, the stone is detained in the urethra, and either produces complete retention of, or additional difficulty in, passing the urine. When it becomes impacted, it is soon surrounded by a tumefaction or exudation of the mucous membrane, while a copious discharge may take place from the urethra. The obstacle to the flow of urine is more dependent upon the form than the size of the calculus; and, the urine finding a passage only along one of its surfaces, the urethra usually becomes distorted. If the stricture is not very close, the calculus may sometimes be displaced by a bougie, and the urine allowed to flow, but this is difficult when it becomes impacted behind a close stricture, in which case the small twisted bougie will oftener insinuate itself between the wall of the urethra and the stone than any other. The calculus sometimes long remains stationary; but when inflammation seizes any of the urinary organs, it enlarges from the deposition of phosphates. Dr. L. gives the very questionable advice to remove it by crushing it with the lithotribe instead of extracting it by external incision.

2. *Gleet.*—The discharge which strictures give rise to at first proceeds from the phlogosis of the mucous membrane situated posteriorly to them; but sooner or later the whole canal participates in this condition. In most cases this effect disappears after the cure of the stricture, however long it may have been present. Very frequently the urethro-prostatic discharges have been mistaken for spermatorrhœa. Nevertheless, the stagnation of the urine posterior to the stricture gives rise to phlegmasia and dilatation of the ejaculatory canals, whence may result a flux of seminal fluid. Numbers of substances, both of the vegetable and mineral kingdoms, have been employed to arrest these discharges. They operate not only as astringents of the mucous follicles, but also by coagulating the albumen which forms so large a portion of the fluids discharged from the urethra. It is important to observe that the sulphates of copper, of zinc, and of alum, which coagulate the albumen when used in moderate proportions, re-dissolve it when employed too strong, and thus may seem to keep up a discharge they first diminished. The nitrate of silver, in small doses, coagulates the albumen; in large ones, it combines with and cauterizes the tissues. Every one knows that nitrate of silver will often suppress these discharges, but it should also be known that, when used to this end, it sometimes induces inflammation and abscess of the prostate. Employed in the large proportions it so often is at present, it often lays the foundation for stricture. Where the nitrate is required, Dr. L. prefers making a slight application of it in substance; but frequently finds this unnecessary by employing the following ointment: Kino 10 parts, Sulph. Zinc. 1 part, Lard 20 parts.

"It is important to observe that these urethral discharges, especially those in which the prostatic fluid prevails, are sometimes accompanied by weakness of the limbs, almost paraplegia, giddiness, dulness of intellect, and all the symptoms M. Lallemand has noted as usually accompanying spermatorrhea, although neither daily or nocturnal pollutions have occurred, or a minute microscopic examination been able to detect a single zoosperm. Further, an engorged state of the prostate without any prostatic discharge, may give rise to this same enfeeblement of the cerebro-spinal system; and, to meet an objection that may be drawn from M. Rayer's work, I will add, in a good number of these cases, there is no symptom of nephritis, and that the thick and puriform condition of the urine disappears when the bladder is emptied by the catheter several times a day—a proof that the deposit does not proceed from the kidneys. * * * * * * * * On the other hand, I may observe, that well-proved seminal emissions may, in some cases, become almost continuous, and last during several years without impairing the general health, the muscular energy of the lower limbs, or the intellectual powers. I have been able several times, by passing a few drops of rosemary or lavender oil down to the vicinity of the neck of the bladder several days in succession, to arrest these seminal discharges, and strengthen the genital organs."

3. *Infiltration of Urine.*—Examples of fatal effusion of urine as a consequence of stricture are unfortunately but too frequent. The delay with which patients seek assistance, deceived often by the fallacious hopes which urine running from the urethra by regurgitation gives them, is usually the cause of this. When we find extensive destruction of the urethra, which often takes place in a latent manner, we must not think that such large aperture has been made at once; it results rather from a destructive gangrene, which destroys and dissects out all the neighbouring parts to which the urine gains access. Incisions into the infiltrated parts must be largely and promptly made, and where the infiltration has been gradual, tending to the production of abscess rather than gangrene, they may arrest the mischief.

"There is one region of the urethra where this slow and successive infiltration puts on quite a peculiar character. I speak of the bulb, and the spongy portion surrounded by the bulbo-cavernosi. More adherent at their edges than at their centre, and surrounded by the sheath forming the superficial fascia of the perinæum, these muscles are raised at their point of contact by the swelling of the indurated cellular tissue into which urine has been infiltrated little by little. Confined at the sides, and finding less resistance at the middle, the increase takes place in the centre and below, giving rise to a kind of crest or *vomer*, which extends from the bulb, having a narrow base of an inch, and a projection of about two inches. These characteristic crests sometimes undergo resolution after the occlusion of the fissure of the urethra, through which the urine oozed out. At other times, after remaining stationary during one or several months, the induration softens at some point, and a succession of small abscesses form, discharge, and close up, without giving issue to a single drop of urine. If by bougies we can re-establish the integrity of the urethra, this indurated tissue gradually undergoes resolution. Sometimes the reproduction of the stricture entails each time a re-opening of the fissures, and a new formation of this crestlike infiltration. Sometimes the indurated tissue rapidly suppurates, but this is rare, and only occurs when, in neglected stricture, the perforation has become much enlarged, and the infiltration more abundant."

4. *Urinary Abscess.*—These are of two kinds; those which result from small and slow infiltrations of urine, and others which do not, at least originally, communicate with the urethra, but result from some external cause or the extension of inflammatory action from within. The last may be induced by the too-long retention of catheters in the urethra during treatment of stricture: by the too sudden increase of the size of the bougie: by cauterization, especially when the caustic comes in contact with the healthy portion of the canal: by the impaction of a portion of calculus in the urethra; and by the peculiar local sensibility of some patients. All these abscesses, from whatever cause proceeding, should be promptly opened; but the catheter, which should be introduced when there is fistulous communication with the urethra, must be abstained from when there is not.

5. *Urinary Fistulæ.*—Sometimes the external orifice of an urinary fistula is placed very far from the perforation of the urethra, and a case is related in which it was placed at the upper-third of the thigh. It is rare to find the urine at first escaping from the urethra by several apertures, but in the course of time, as many as ten or twelve may be observed in some patients. The amount of urine passed by the fistula varies much, being occasionally the entire quantity, and in others a mere oozing. It is not uncommon to find calculous deposits in the track of the fistula, or in the urethra, just before the fistula is given off. As a general rule, the fistulæ are best treated by leaving a catheter in the urethra, but some are not only not cured, but are aggravated by this measure—the aperture closing as soon as the instrument is removed. The calculi which may form must be extracted, and M. L. refers to a case, in which 22 had to be removed, each of which was enclosed in a little sac requiring separate incision.

This chapter contains an appendix, detailing some new operative procedures for the relief of *Vesico-Vaginal Fistulæ.*

M. Lallemand's observations upon the Venereal Disease do not seem to us of sufficient novelty or importance to call for attention; and we shall defer noticing those upon Diseases of the Prostate until we are in possession of Dr. Leroy's work on that subject, which he promises shortly.

Dr. Leroy's Treatise is abundantly illustrated by woodcuts and lithographs, exhibiting some of the more interesting lesions, and his endless multiplicity of instruments; but the absence of all explanatory references is a great defect.

We regret to find this author interspersing his valuable work with so many severe observations upon certain of his *confreres.* It is true the attack has come from their side, the sore point being the old quarrel concerning "Specialism" in surgery. We are happy to say that the recriminations, accusations, and harsh epithets, so freely bandied about among the Parisian medical men of note and reputation, are never employed by the same high class of practitioners, and scarcely ever by any class whatever, in our own country.

